# Molecular and Phenotypic Characterization of *Aerococcus viridans* Associated with Subclinical Bovine Mastitis

**DOI:** 10.1371/journal.pone.0125001

**Published:** 2015-04-28

**Authors:** Gang Liu, Yongxia Liu, Tariq Ali, Miro Ferreri, Jian Gao, Wei Chen, Jinhua Yin, Jingliang Su, Séamus Fanning, Bo Han

**Affiliations:** 1 Department of Clinical Veterinary Medicine, College of Veterinary Medicine, China Agricultural University, Beijing, P.R. China; 2 UCD-Centre for Food Safety, School of Public Health, Physiotherapy & Population Science, University College Dublin, Belfield, Dublin 4, Ireland; The University of Hong Kong, HONG KONG

## Abstract

*Aerococcus viridans* is a wide spread bacterium in the environment and clinically this organism is associated with different diseases in animals and humans. However, the geno- and phenotypic characterization of *A*. *viridans* associated with bovine mastitis has not yet been reported. The objectives of this study were to investigate the genetic and phenotypic diversity of *A*. *viridans* isolates using three different molecular methods including 16S rRNA gene sequencing, pulsed-field gel electrophoresis and random amplified polymorphic DNA (RAPD) along with biochemical tests, including antimicrobial susceptibility test. In total, 60 *A*. *viridans* strains were cultured from dairy herds presenting with subclinical mastitis. The results of biochemical tests revealed that most of the isolates (75.0%) were accurately identified by API Rapid 20 Strep system and the majority of *A*. *viridans* strains (96.7%) were found to be catalase negative, while two (3.3%) isolates were weakly positive. All isolates were resistant to trimethoprim-sulfamethoxazole, followed by streptomycin (96.7%), tetracycline (65.0%) and clindamycin (56.7%) by minimum inhibition concentration-determining broth microdilution technique. As compared to the sequence of 16S rRNA gene, both PFGE and RAPD showed their capacities to discriminate the intra-species diversity of *A*. *viridans*. Furthermore, most of the isolates obtained from the same herd or region belonged to the same major RAPD group, which indicated that RAPD is an appropriate assay for tracking the origins of isolates and epidemiological studies of *A*. *viridans*. This is a novel approach to use three molecular techniques and to compare their efficiency regarding the genetic diversity of *A*. *viridans*. The data suggest that *A*. *viridans* associated with subclinical mastitis has a considerable phenotypic and genotypic diversity.

## Introduction

*Aerococcus viridans* is a gram positive coccus, which belongs to the family of Streptococcaceae under the genus of *Aerococcus* [1]. Phenotypically, *A*. *viridans* is an oxidase-negative, catalase-negative or weakly-positive, facultative anaerobic bacterium [2], and usually shows green alpha hemolysis on blood agar [3]. The aeroccocci may easily be misidentified as staphylococci or streptococci, and this may be the reason that the incidence of infections caused by aerococcal spp. has been underestimated [4].

*A*. *viridans* is wide spread organism in the environment [2], but it has been clinically associated with different human and animal infections [5,6]. In veterinary medicine, *A*. *viridans* has been often associated with aquatic animal infections, like gaffkemia in lobster [7], septicemia in sea turtles [8] and mortality in tilapia [9]. Furthermore, *A*. *viridans* was isolated from swine arthritis, meningitis and pneumonia [10]. In dairy industry, *A*. *viridans* is associated with bovine severe respiratory syndrome [11], and has also been isolated from the milk of cows with subclinical and clinical mastitis [12–14].

Mastitis is an important problem affecting dairy cattle and constitutes a source of huge economic losses for the dairy industry due to the detrimental effects on milk quality and yield [15]. Although some previous studies reported the isolation of *A*. *viridans* from mastitic milk samples [12–14], the published information on molecular characterization of the bacteria from bovine mastitis is scarce, especially data from China. Moreover, a tool of accurate discrimination and tracking of *A*. *viridians* isolated from milk is not yet clearly established, as well as a lack of knowledge regarding the genetic diversity of the strains, their biochemical and antimicrobial susceptibility profiles.

The present study was designed with the core objective to investigate the phenogenetic diversity of *A*. *viridans* isolates associated with subclinical mastitis in dairy farms in Northern China. Therefore, the isolated *A*. *viridans* were defined phenotypically with their biochemical profiles and antimicrobial susceptibility. Furthermore, three different techniques: 16S rRNA gene sequencing, RAPD and PFGE analysis were applied and compared in their efficiency in discriminating and tracking *A*. *viridans*.

## Materials and Methods

### Ethics

Milk samples were collected from animals with subclinical mastitis with the prior consent of farm owners and under the ethical approval granted by College of Veterinary Medicine, China Agricultural University. The protocol was permitted by the owners of the dairy farms and all efforts were made to minimize cows suffering.

### Sample collection

From April 2010 to December 2013, milk samples (*n* = 1,008) were obtained from Chinese Holsteins lactating cows suffering from subclinical mastitis belonging to major dairy farms in the Northern China (Beijing = 256, Tianjin = 220 and Hebei = 532 samples). Subclinical mastitis was determined when the somatic cell counts (SCC) ≥500,000 cells/ml (Fossomatic 5000TM, Foss Electric, Hillerød, Denmark), with decreased milk production and without visual inflammation of the udder [16]. Following, the teat was disinfected with 70% ethanol and the first three streams were discarded and quarter milk samples were aseptically collected in sterile tubes, cooled and transported to the laboratory [17].

### Isolation and identification of the bacterial isolates

Milk samples (50 μl) were streaked on TSA (Trypticase Soya Agar; Sigma, India) supplemented with 5% defibrinated sheep blood (hereafter SBA, Sheep Blood Agar) and incubated aerobically at 37°C for 24 h. Following, all small (~1 mm), non-pigmented or yellow pigment colonies with alpha hemolytic activity were chosen for further identification [1]. Suspected colonies were picked, purified and primarily identified on the basis of conventional methods including colony morphology, Gram staining and hemolytic activity [1]. Confirmation of the suspected isolates was conducted by amplification of 750-bp fragments of 16S rRNA gene, with a pair of universal primers [18]. The PCR products were purified and sequenced on an ABI 3730 automated sequencer at Beijing Sunbiotech, Inc. (Beijing, China). The sequence data were compared with the GenBank database using BLAST software and homology level ≥98% was considered adequate for species identification. All *A*. *viridans* isolates were stored in brain heart infusion broth (BHI; Invitrogen, Beijing, China) with 20% glycerol at -80°C until tested.

### Biochemical tests

A commercially available identification system API Rapid 20 Strep system (bioMérieux, SA, Marcy l’Etoile, France) were used to achieve biochemical profiles of all *A*. *viridans* isolates according to the manufacturer’s instructions. Moreover, all of the isolates were tested for oxidase, catalase and the tolerance test in 6.5% and 10% NaCl [1]. In addition, the identification efficiency of API Rapid 20 Strep Strip was evaluated. Isolate identification to the species level was divided into four subgroups: (i) excellent species identification, %id of ≥99.9% and a *T* value of ≥0.75; (ii) very good species identification, %id of ≥99.0% and a *T* value of ≥0.5; (iii) good species identification, %id of ≥90.0% and a *T* value of ≥0.25; and (iv) acceptable species identification, %id of ≥80.0% and a *T* value≥0.0 (with %id and *T* being manufacturer-defined variables) [19].

### Antimicrobial susceptibility

The *in vitro* susceptibility of all *A*. *viridans* isolates were determined by standard microdilution method according to the guideline of Clinical and Laboratory Standards Institute [20], using Mueller-Hinton broth supplemented with 5% lysed horse blood. The antibiotics tested were: penicillin, ampicillin, ceftiofur, erythromycin, clindamycin, chloramphenicol, tetracycline, kanamycin, gentamicin, trimethoprim-sulfamethoxazole, streptomycin, vancomycin, ciprofloxacin and norfloxacin. Since no specific MICs breakpoints for aerococci are available, the MICs breakpoints used for resistance to penicillin, ampicillin, ceftiofur, clindamycin, chloramphenicol, tetracycline, kanamycin and gentamicin were those recommended by the CLSI [20] for testing animal streptococci other than *S*. *pneumonia*. However, for vancomycin and streptomycin, the MICs breakpoints were those recommended for testing animal enterococci [20]. For ciprofloxacin and norfloxacin, the MICs breakpoints were those used for testing enterococci [21]. For erythromycin and trimethoprim-sulfamethoxazole, the breakpoints were those recommended for animal streptococci and streptococcus pneumonia, respectively. *S*. *aureus* ATCC 27923 was used as the reference strain for quality control.

### DNA purification

For 16S rRNA gene sequencing and RAPD test, the bacterial DNA was extracted as described previously [16]. The DNA concentration of the supernatants was measured using a Nanodrop ND-1000 spectrophotometer (Thermo scientific, USA) and adjusted to be 100 ng/μl approximately.

### Phylogenetic analysis based on 16S rRNA gene

For sequence analysis, a 1400-bp fragment of 16S rRNA gene of all *A*. *viridans* isolates was amplified and sequenced as previously described [9]. The PCR products were purified and sequenced on an ABI 3730 automated sequencer at Beijing Sunbiotech Inc., Beijing. The obtained chromatographs were verified by eye and the sequences were aligned using the program MUSCLE [22] as implemented in MEGA5 [23]. The program DNAsp v.5 [24] was used to compute haplotype (h) [25] and nucleotide (π) [26] diversity.

### Analysis of the chromosomal DNA restriction profiles by PFGE

For PFGE analysis, Chromosomal DNA of *A*. *viridans* isolates was extracted in agarose plugs, treated with SmaI restriction endonuclease, and analyzed by pulsed-field gel electrophoresis (PFGE) as previously described [7]. The PFGE patterns were visually assessed and Gel images were analyzed using InfoQuest FP software (Bio-Rad Laboratories, USA). Group analysis was carried out by using the Dice coefficient and the unweight pair group method with arithmetic averages (UPGMA) of the PFGE profiles. *A*. *viridans* isolates were assumed to belong to the same group if the Dice correlation coefficient was 80% or greater, according to Tenover's criterion [27]. Groups composed by three or more isolates were named by alphabetical letters.

### Analysis of DNA amplification products obtained by RAPD

To carry out RAPD analysis, 15 arbitrary 10-bp primers were synthesized and used. In short, the bacterial genomic DNA of *A*. *viridans* isolates (50 ng) was subjected to PCR with each of the 15 arbitrary primers. The primers which generated reproducible patterns with an appropriate number of amplified products were considered for analysis [28]. The RAPD reaction was performed in a 50 μl final volume containing 0.25 μl of DNA polymerase, 10 μl of 5×PrimeSTAR Buffer, 4 μl of dNTP Mixture (Takara, Japan), 50 pmol of arbitrary primer, 100 ng of template DNA. The procedure of amplification was as follows: initial denaturation at 95°C for 5 min; 35 cycles of 1 min denaturation at 95°C, 60 s annealing at 36°C, 2 min extension at 72°C; and final extension for 5 min at 72°C [29]. Products of amplification were separated by electrophoresis in a 1.5% agarose gel stained with ethidium bromide and then photographed under UV light. The RAPD amplification profiles were initially compared by visual inspection, followed by computer-assisted analysis performed as described above for analysis of the PFGE profiles. Isolates showing 80% similarity or higher were assumed to belong to the same group [30].

### Discriminatory power of genotyping methods

A discrimination index was used to assess the discriminatory power of 16S rRNA gene sequencing, RAPD and PFGE for genotyping *A*. *viridans* isolates as described before [31]. The discrimination index (D) is given by the following equation:

*D* = 1 - [1/*N* (*N*—1)] ∑*nj* (*nj*—1), where *N* is the total number of isolates in the sample population, and *nj* is the number of isolates belonging to the *j*th type.

## Results

### Isolation and identification of *A*. *viridans*

A total of 1,008 milk samples were collected from cows with subclinical mastitis from 19 different dairy farms. *A*. *viridans* isolates were recovered from 60 cows (one isolate per cow) on 10 farms located in Hebei province and two municipalities (Beijing and Tianjin). In total, 60 *A*. *viridans* isolates were identified and analyzed (Table 1). Furthermore, information of all isolates identified by 16S rRNA sequence is shown in S1 Table.

**Table 1:** Details of *A*.*viridans* strains isolated from subclinical bovine mastitis.

Isolates	Herd Code	Sampling site	No. of Isolates	No. of Samples	Isolation Rate	Year of Isolation
*A*.*V*. 01- *A*.*V*. 10	HB-L	Langfang City, Hebei	10	34	29.4%	2010
*A*.*V*. 11- *A*.*V*. 16	HB-T	Tangshan City, Hebei	6	41	14.6%	2013
*A*.*V*. 17- *A*.*V*. 23	HB-Z	Zhangjiakou City, Hebei	7	51	13.7%	2011
*A*.*V*. 24- *A*.*V*. 27	HB-S	Shijiazhuang City, Hebei	4	48	8.3%	2010
*A*.*V*. 28- *A*.*V*.36	BJ-H	Haidian District, Beijing	9	39	23.0%	2010
*A*.*V*. 37- *A*.*V*.39	BJ-S	Shunyi District, Beijing	3	55	5.4%	2012
*A*.*V*. 40- *A*.*V*.45	BJ-T	Tongzhou District, Beijing	6	26	23.1%	2012
*A*.*V*. 46- *A*.*V*.54	TJ-X	Xiqing District, Tianjing	9	44	20.5%	2013
*A*.*V*. 55- *A*.*V*.57	TJ-J1	Jingnan District, Tianjing	3	52	5.8%	2010
*A*.*V*. 58- *A*.*V*. 60	TJ-J2	Jingnan District, Tianjing	3	36	8.3%	2011

### Biochemical tests

The results of API 20 Strep system were taken after 24 h of incubation at 37°C. In total, 10 different numerical codes were achieved. All of the strains showed positive results to HIP (hippuric acid hydrolysis), PYRA (pyrolidonylarlyamidase), LAC (lactose fermentation) and TRE (trehalose fermentation) tests. None of the strains can deaminate arginine. Most of the strains showed positive results to Voges-Proskauer tests. Details of the numerical codes and discrepant tests obtained are shown in Table 2. With regards to catalase activity, *A*. *viridans* isolates showed two patterns. Fifty eight (967%) isolates were catalase negative, however, two (3.3%) isolates were weak positive. Furthermore, all of *A*. *viridans* isolates showed negative results for oxidase test and they tolerated 6.5% and 10% NaCl. In addition, a total of 45 (75.0%) tested isolates were accurately identified (excellent to good identification) by using this identification system, while 15 (25.0%) isolates were only identified to *Aerococcus* spp.

**Table 2:** Results of API 20 Strep system for 60 *A*. *viridans* strains.

Numerical code	Identification result (No. of isolates)	Comments	Discrepant biochemical tests^*a*^
7300450	*A*. *viridans*Ⅲ (7)	good species identification	VP, MAN
7300550	*A*. *viridans* (8)	excellent species identification	VP
2502410	*A*. *viridans* (2)	good species identification	βGUR, RIB
7100450	*Aerococcus* spp. (5)	NA^b^	VP, αGAL, MAN, RAF
7100550	*A*. *viridans* (4)	good species identification	VP, αGAL
6700410	*Aerococcus* spp. (10)	NA	αGAL, βGUR, MAN, RAF
6700550	*A*. *viridans* (5)	excellent species identification	None
7700450	*A*. *viridans* (8)	excellent species identification	VP, MAN
7300050	*A*. *viridans* (1)	good species identification	VP, MAN, LAC
7300150	*A*. *viridans* (10)	very good species identification	VP, LAC

^a^MAN, LAC, RAF and RIB correspond to the acidification of mannitol, lactose, raffinose and ribose, respectively; αGAL and βGUR correspond to the production of α-galactosidase and β-glucuronidase, respectively; VP, corresponds to acetoin production (Voges-Proskauer test).

^b^NA, not applicable.

### Antimicrobial susceptibility

Antimicrobial susceptibility profiles of all *A*. *viridans* isolates for twelve antibiotics were determined by standard microdilution method (Table 3). Fifty seven (95.0%) isolates were susceptible to β-lactam antibiotics (penicillin, ampicillin and ceftiofur). However, lower frequency of susceptibility was recorded to tetracycline, clindamycin, streptomycin and trimethoprim-sulfamethoxazole, and more than 56.7% of the isolates being resistant to these antibiotics. Furthermore, the MIC_50_ (MIC at which 50% of isolates are at or below) for trimethoprim-sulfamethoxazole and streptomycin was 128 μg/ml, and the MIC_90_ (MIC at which 90% of isolates are at or below) was ≥elo μg/ml. The resistance rates of *A*. *viridans* to the other antibiotics tested was variable, ranging from 1.7% (vancomycin) to 40.0% (norfloxacin). Whereas, three isolates (5.0%) which were *A*.*V*. 47, *A*.*V*. 49 and *A*.*V*. 50, showed resistance to all tested antibiotics except tetracycline, kanamycin, ciprofloxacin and vancomycin.

**Table 3:** Minimum inhibition concentrations (MICs) distribution and resistance rates of 60 *A*. *viridans* strains.

	No. of isolates for which the MIC (mg/L) were:														
**Antimicrobials**	≤0.12	0.25	0.5	1	2	4	8	16	32	64	128	≥256	**MIC** ^**a**^_**50**_	**MIC**_**90**_	**R.S** ^**b**^ **%**
Penicillin	25	19	5	6	2	**0** ^**c**^	0	0	1	2	0	0	0.25	1	5.0
Ampicillin	32	17	1	2	1	3	**0**	0	0	0	1	2	⩽0.12	4	5.0
Ceftiofur	18	30	2	1	1	5	**0**	0	0	2	1	0	0.25	4	5.0
Erythromycin	11	13	17	**8**	4	3	1	0	1	2	0	0	0.5	4	31.6
Clindamycin	3	8	4	5	6	**12**	10	4	3	2	3	0	4	32	56.7
Chloramphenicol	1	4	3	4	5	12	8	**6**	3	7	3	4	8	128	38.3
Tetracycline	0	2	3	5	4	7	**16**	5	8	2	5	3	8	128	65.0
Kanamycin	0	0	2	2	6	7	18	13	8	**2**	1	1	8	32	6.7
Gentamicin	0	5	3	8	9	14	13	**3**	2	3	0	0	4	16	13.3
Vancomycin	0	6	5	4	7	10	19	8	**0**	1	0	0	4	16	1.7
Ciprofloxacin	21	14	8	10	3	**4**	0	0	0	0	0	0	0.25	2	6.7
Norfloxacin	0	14	10	5	2	5	0	**10**	8	6	0	0	2	32	40.0
Streptomycin	0	0	0	0	0	0	0	0	2	**12**	32	14	128	≥256	96.7
SXT^**d**^	0	0	0	0	0	**0**	0	0	0	3	28	29	128	≥256	100

^**a**^MIC, Minimum inhibition concentrations

^**b**^R.S, Resistant strains

^**c**^The number of isolates for each antimicrobial agent MICs breakpoint is highlighted by bold text.

^**d**^SXT, Trimethoprim-sulfamethoxazole

### Phylogenetic analysis based on 16S rRNA gene

According to the results of 16S rRNA gene sequencing, among the whole data set (n = 60), 57 isolates belonged to the dominant clade (Haplotype 1) and shared the same sequence data deposited in GenBank under the accession number of KM096431. The remaining three isolates were grouped into another clade (Haplotype 2) and also shared the same sequence data deposited in GenBank under the accession number of KM096432. Data set diversity indices revealed low levels of haplotype diversity in *A*. *viridans* (Hd: 0.097) and low nucleotide diversity (Pi: 0.00104). The average number of nucleotide differences (*k*) was 1.449.

### Analysis of the chromosomal DNA restriction profiles by PFGE

**Fig 1:**
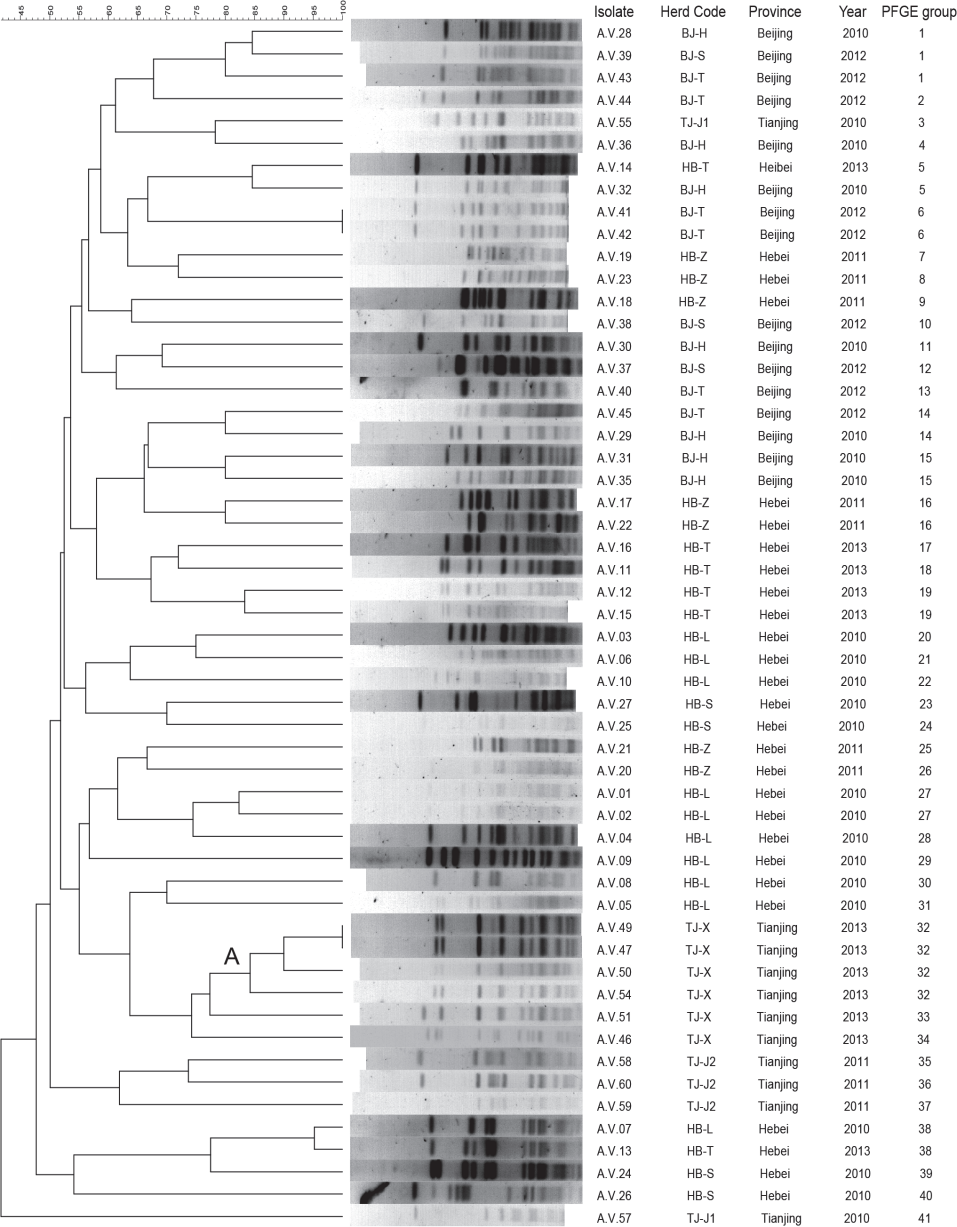
Dendrogram resulting from a computer-assisted analysis of the PFGE profiles of *A*. *viridans* isolates recovered from bovine subclinical mastitis. The Dice coefficient and a tolerance of 1.5% were used for calculating the similarities and clustering among the profiles.

The genetic characterization of *A*. *viridians* isolates was achieved by PFGE with the restriction enzyme SmaI. The restriction patterns of the same isolate generated was found to be stable and reproducible in three different trials. The majority of isolates (93.2%) were successfully characterized. In total, fifty two PFGE profiles were identified and forty one groups were detected by PFGE analysis among 60 isolates (Fig 1). Overall, according to the results of PFGE, *A*. *viridans* isolates showed high genetic diversity, with 31 (75.6%) PFGE groups composed by single isolates and 8 (19.5%) by double isolates. Furthermore, group 1 was consisted of 3 strains from different farms in Beijing and group 2 was comprised of 4 strains and all of them were recovered from Herd TJ-X in Tianjing in the same year. Only two pairs of isolates (*A*. *viridans* 41, 42 and 47, 49) were recovered in the same year from different cows at the same farm exhibited undistinguishable PFGE profiles.

### Analysis of DNA amplification products obtained by RAPD

**Fig 2:**
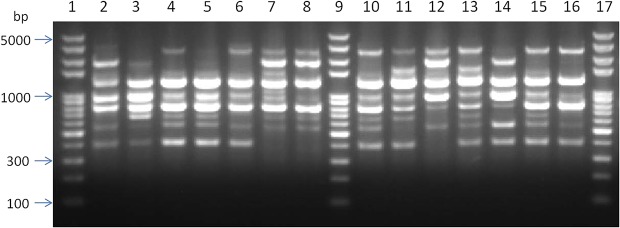
Representatives of RAPD fingerprints identified among *A*. *viridans* isolates from subclinical mastitis. Lanes 1, 9, 17 molecular size markers (in base pairs; DNA ladder ranging from 100 to 5,000 bp); Lane 2 *A*.*V*. 39 (RAPD group 9, BJ-S, 2012); Lane 3 *A*.*V*. 38 (RAPD group 9, BJ-S, 2012); Lane 4 *A*.*V*. 31 (RAPD group 6, BJ-H, 2010); Lane 5 *A*.*V*. 45 (RAPD group 6, BJ-T, 2012); Lane 6 *A*.*V*. 29 (RAPD group 6, BJ-H, 2010); Lane 7 *A*.*V*. 51 (RAPD group 8, TJ-X, 2013); Lane 8 *A*.*V*. 48 (RAPD group 8, TJ-X, 2013); Lane 10 *A*.*V*. 30 (RAPD group 6, BJ-H, 2010); Lane 11 *A*.*V*. 24 (RAPD group 7, HB-S, 2010); Lane 12 *A*.*V*.43 (RAPD group 14, BJ-T, 2012); Lane 13 *A*.*V*. 28 (RAPD group 6, BJ-H, 2010); Lane 14 *A*.*V*. 41 (RAPD group 13, BJ-T, 2012); Lane 15 *A*.*V*. 33 (RAPD group 6, BJ-H, 2010); Lane 16 *A*.*V*. 53 (RAPD group 6, TJ-X, 2013).

**Fig 3:**
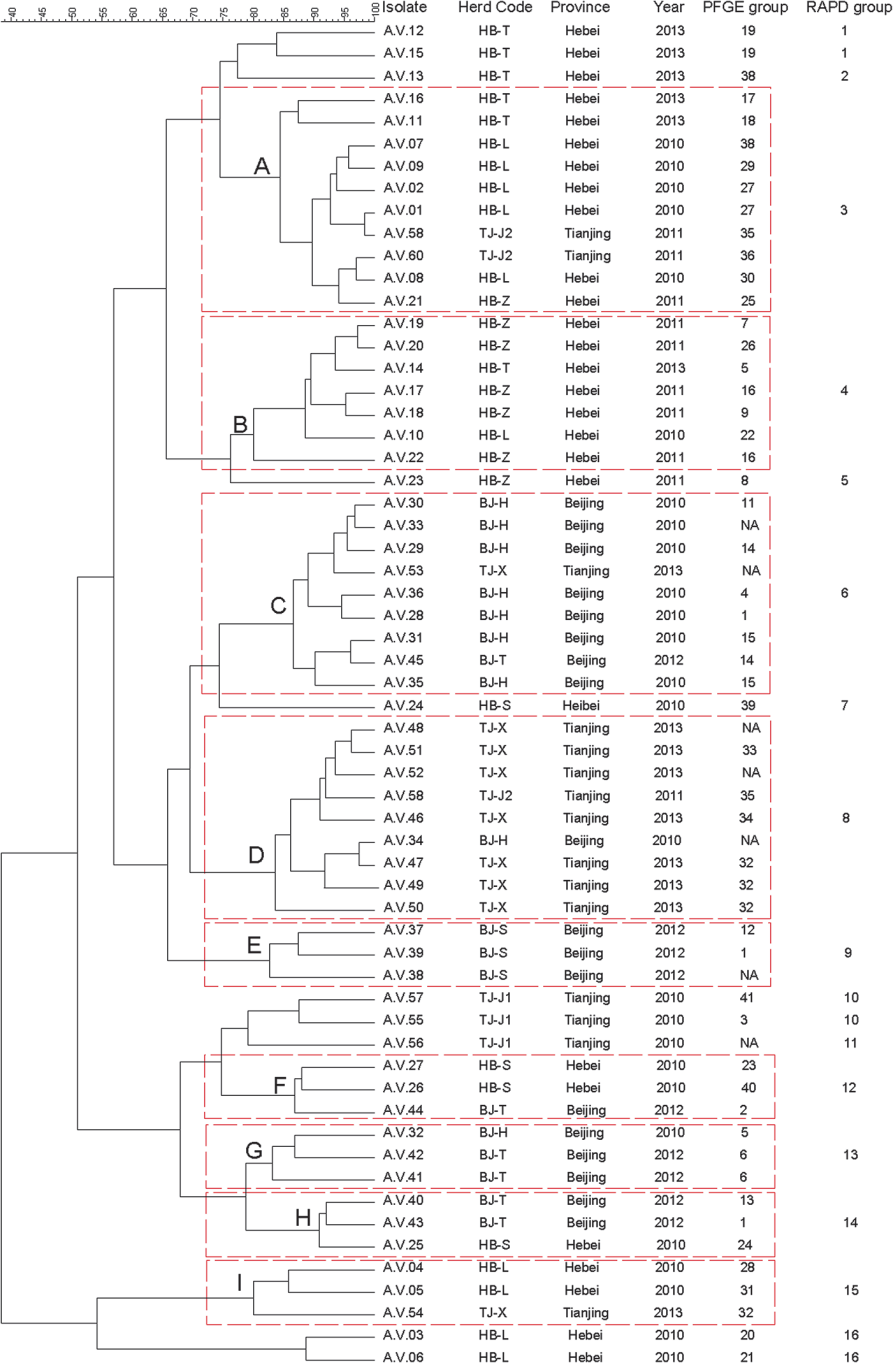
Dendrogram resulting from a computer-assisted analysis of the RAPD profiles of *A*. *viridans* isolates recovered from bovine subclinical mastitis. The Dice coefficient and a tolerance of 1.5% were used for calculating the similarities and clustering among the profiles. NA, PFGE method was not applicable for the characterization of this isolate according to the results of PFGE in our study.

The RAPD analysis of the *A*. *viridans* was performed on 30 randomly selected strains using 15 different primers. Only primer S201 (5’-GGG CCA CTC A-3’) generated reproducible patterns with an appropriate number of amplified products. The selected primer was then used to analyze 60 isolates of *A*. *viridans*. The reproducibility of the results was verified by repeating all RAPD reactions for each strain for three times. No considerable differences were observed in the profiles, although some bands varied in intensity. The example of banding patterns for primer 201 from 14 selected strains is presented in Fig 2. The relationships among RAPD profiles of the 60 *A*. *viridans* isolates are shown in the dendrogram (Fig 3). The 60 *A*. *viridans* isolates were divided into 16 RAPD groups with percentages of similarity ranging from approximately 38–98%. The majority of isolates in major RAPD groups were originating from the same herd or origin. A, B, C and D were the main groups and each group was consisted of at least seven strains in the dendrogram. The strains of group A and B were dominated by strains from Hebei province. Whereas, most of the strains in group C and D were isolated from Beijing and Tianjing, respectively. Furthermore, group E was only comprised of the strains from Herd BJ-S.

### Discriminatory power of genotyping methods

The discrimination indexes obtained for the three genotyping methods were 0.097 (16S rRNA gene sequencing), 0.988 (PFGE) and 0.912 (RAPD).

## Discussion

*Aerococcus viridans* has been isolated from the milk of cows suffering from subclinical and clinical mastitis [12–14], but has rarely been investigated as the main objective. In the present study we investigated the phenotypic characterizations and the phylogenetic diversity of 60 *A*. *viridans* isolates associated with subclinical mastitis in the dairy farms of Northern China.

With respect to the isolation and identification of *A*. *viridans*, the green alpha hemolysis on blood agar, and the results of Gram staining showed gram-positive cocci arranged in tetrads, makes this organism unique and different from most of the other species. Furthermore, biochemically, *A*. *viridans* isolates showed two different patterns to catalase activity, the majority of isolates (96.7%) were found negative, while two isolates (3.3%) were found weak positive. This indicates that the catalase test cannot be used as a primary step test for identification of *A*. *viridans*. A wide variety of biochemical profiles was observed according to the different numerical codes given by the API 20 Strep system. In a previous description, *A*. *viridans* isolates were negative to Voges-Proskauer test [1]. However, most of isolates in the present study showed positive results to the Voges-Proskauer test. The reason might be attributed to the phenotypic diversity of the organisms. With regards to the efficiency of identification, the results of the API 20 Strep strips showed that most of the *A*. *viridans* isolates (75.0%) were accurately identified. Whereas, 15 (25%) isolates were only identified to *Aerococcus* spp. This inaccuracy can be expected as it has also been shown that API 20 Strep system is not completely reliable for the accurate identification of aerobic gram-positive catalase-negative cocci [10,19].

According to the previous reports [13,32], *A*. *viridans* isolates from different geographical areas were highly diverse in their antibiotic resistance patterns. Whereas, there is limited published data on antibiotic susceptibility of *A*. *viridans* isolated from bovine mastitis. Špaková et al. [13] reported that *A*. *viridans* isolates from clinical and subclinical cases of bovine mastitis in Slovakia showed a high level resistance to beta lactam antibiotics, but the organisms were susceptible to neomycin and ciprofloxacin; while sporadically resistance to streptomycin and erythromycin was detected. In our study, the results of antimicrobial susceptibility test showed a different pattern from the results of above study, particularly for the resistance patterns of beta lactam antibiotics. All of the isolates were resistant to trimethoprim-sulfamethoxazole, followed by streptomycin (96.7), tetracycline (65.0%) and clindamycin (56.7%). Furthermore, all of the isolates showed a high MIC value to streptomycin and shared the MIC_50_ (128μg/ml) and MIC_90_ (≥256μg/ml) with trimethoprim-sulfamethoxazole. On the contrary, only three (5.0%) *A*. *viridans* isolates were resistant to beta lactam antibiotics including penicillin, ampicillin and ceftiofur, and four (6.7%) isolates showed resistance to ciprofloxacin. The differences in antibiotic resistance patterns may be attributed to different application of antibiotics in Slovak and Chinese veterinary practices and therefore a specific adaption of *A*. *viridans* [33]. However, the resistance pattern of our isolates is similar to the investigation of Owens et al. [32]. According to the latest report [5], an *A*. *viridans* strain which was resistant to vancomycin was isolated from a human patient in the Southern China. While in our study, one isolate showed resistance pattern to vancomycin was also isolated from bovine mastitis in the Northern China. Notably, three (5.0%) isolates were found to be resistant to all tested antibiotics except tetracycline, kanamycin, ciprofloxacin and vancomycin, which mean there exists a small group of this species possessing a strong and multiple resistant pattern.

To achieve knowledge about the phylogeny of strains, several well-established molecular methods could be proposed. The 16S rRNA gene sequencing is a common marker on inter- and intra-phylogenetic level [9]. PFGE is one of the most powerful molecular typing methods and has been successfully applied to a wide range of different bacterial pathogens including *A*. *viridans* [10]. In addition, RAPD analysis is a sensitive PCR-based method that uses arbitrary primers to generate whole genome DNA fingerprints for discriminating between strains [29]. With respect to the genetic diversity of our isolates, the three different molecular typing systems including 16S rRNA gene sequencing, PFGE and RAPD were simultaneously used for the first time to evaluate overall DNA polymorphism. Molecular typing techniques are specifically useful for epidemiological studies, as they provide information on the genetic relatedness of strains, sources of infection, detection of particularly virulent strains and the geographical and host distribution of possible variants of a specific pathogen [34].

The results of 16S rRNA gene sequencing analysis showed that all of the isolates can be divided into two well supported clades, and in each clade only one haplotype was observed. The limited diversity indexes indicate: (a) minimal polymorphism of the 16S rRNA gene of *A*. *viridans*; (b) no phylogenetic pattern in relation to variables according of geography (dairy farms); and (c) very limited discrimination of *A*. *viridans* strains. The results were in accordance with the previous report that comparison of 16S rRNA gene sequences has been recognized as an invaluable tool for confirming bacterial species identity but not for differentiation among strains, since the sequence shows limited intraspecies variation [35].

In the present research, we used RAPD and PFGE assays for the first time to subtype *A*. *viridans* isolates associated with bovine mastitis. Both of the molecular typing methods showed good discriminatory power (as indicated by discriminatory indexes of 0.90), although SmaI DNA restriction profiles could not be obtained for all isolates. The failure of PFGE typing to the minority of *A*. *viridans* has also been documented in previous report [10]. Both methods identified a variety of profiles and groups among the strains, this may suggest that there exist great genetic diversity within strains in Northern China. By contrast, a research about *A*. *viridans* isolates from lobster reported that there was very limited genetic diversity for this species in England and Wales, based on results of PFGE [7]. Similarly, the same phenomenon was also observed for the isolates from lobster in North America, using RAPD and 16S rRNA gene sequence [29].

Furthermore, some discrepancy between the results of RAPD and PFGE analysis was observed. Our results of RAPD showed that several major groups were dominated by isolates originating from the same herd or region, which indicates that there existed a few prevalent strains infecting each herd or area. On the other hand, the results of PFGE showed great genetic diversity to *A*. *viridans* isolates, which indicates there is no clear epidemiological relationship among the strains. When a cutoff of 60% similarity among profiles was applied to analyze the results of PFGE, some major groups from different herds or areas were also detected. This may be attributed to the reason that RAPD is less discriminative than PFGE, which is in accordance with the results of discrimination indexes. Therefore, our findings recommend the importance of using more than a single molecular technique as the basis for assessment of the genetic relationship among strains.

In some strains, there were concordances between the groups obtained with PFGE/16S rRNA gene sequencing and RAPD, such as all of the isolates in the small clade of 16S rRNA gene sequencing were distributed into group I of RAPD. In addition, *A*.*V*.47 (TJ-X), *A*.*V*.49 (TJ-X) and *A*.*V*.50 (TJ-X), which had a strong antibiotic resistant pattern, were placed in the same group in PFGE and RAPD system. This indicates that there existed an epidemiological group which acquired strong antibiotic resistant ability in that dairy herd. On the other hand, the two isolates (*A*.*V*.12, *A*.*V*.15), which showed weak positive reaction to the catalase results, were also assigned to the same group in PFGE and RAPD system. These findings elucidate that the detection of groups of higher genetic similarities composed of the isolates which may harbor the same phenotype; this is in accordance with the previous report [36]. Therefore, epidemiological studies of *A*. *viridans* could be useful to detect the phenotype of the species including antibiotic resistant patterns to guide the clinical work.

In our study, some prevalent RAPD groups were observed in different areas. While group A, which was prevalent in Hebei province and group C, which was prevalent in Beijing, also existed in dairy farms in Tianjing. In addition, Group D, which was prevalent in Tianjing, was also detected from dairy farms in Beijing. Considering the close relatedness of these three areas, this phenomenon can be explained as a result of the commercial movement of infected animals between these areas. Furthermore, several different RAPD groups were obtained from the same dairy farms, which may indicate the existence of several strains originating from several sources of infection in one dairy farm.

In summary, *A*. *viridans* isolates from bovine subclinical mastitis in Northern China showed great diversity with regard to phenotypic characterization. Although a few major groups represented by highly related isolates were detected, genotyping by either PFGE or RAPD displayed a variety of profiles, indicating the substantial genetic diversity of *A*. *viridans* strains isolated from bovine mastitis. Our study concluded that mastitis associated with *A*. *viridans* in different areas is due to the distribution of various isolates rather than the epidemic spread of a single strain. The comparison of three molecular typing methods showed that RAPD analysis is an excellent tool for molecular typing, along with possessing brilliant discriminative ability and tracking origins of the isolates, which can be used as a rapid method of comparing *A*. *viridans* strains for epidemiological investigation. To the best of our knowledge, this is the first report of isolation, identification, antibiotic sensitivity and genetic diversity of *A*. *viridans* associated with bovine subclinical mastitis from China.

## Supporting Information

S1 Table Identification data of isolates by 16S rRNA sequence. (DOCX)
