# Supplementary material for: Molecular and Phenotypic Characterization of Aerococcus viridans Associated with Subclinical Bovine Mastitis
Source: PLoS One. 2015 Apr 28;10(4):e0125001. doi: 10.1371/journal.pone.0125001 (PMC4412496; doi:10.1371/journal.pone.0125001)
Supplement: S1 Table — (DOCX) [file pone.0125001.s001.docx]

**S1 TABLE. Identification data of isolates by 16S rRNA sequence.**

| Species | Isolate number | Percentage (%) |
| --- | --- | --- |
| *Streptococcus uberis* | 112 | 35.8 |
| *Streptococcus dysgalactiae* | 78 | 24.9 |
| *Aerococcus viridans* | 60 | 19.2 |
| *Trueperella pyogenes* | 38 | 12.1 |
| *Enterococcus faecalis* | 25 | 8.0 |
| In total | 313 | 100% |
